# Impact of ^18^F-FDG PET/CT in Staging Patients With Small Cell Lung Cancer: A Systematic Review and Meta-Analysis

**DOI:** 10.3389/fmed.2019.00336

**Published:** 2020-01-29

**Authors:** Francesco Martucci, Mariarosa Pascale, Maria Carla Valli, Gianfranco A. Pesce, Patrizia Froesch, Luca Giovanella, Antonella Richetti, Giorgio Treglia

**Affiliations:** ^1^Clinic of Radiation Oncology, Oncology Institute of Southern Switzerland, Ente Ospedaliero Cantonale, Bellinzona, Switzerland; ^2^Clinical Trial Unit, Ente Ospedaliero Cantonale, Bellinzona, Switzerland; ^3^Clinic of Medical Oncology, Oncology Institute of Southern Switzerland, Ente Ospedaliero Cantonale, Bellinzona, Switzerland; ^4^Clinic of Nuclear Medicine, Imaging Institute of Southern Switzerland, Ente Ospedaliero Cantonale, Bellinzona, Switzerland; ^5^Department of Nuclear Medicine and Molecular Imaging, Lausanne University Hospital and University of Lausanne, Lausanne, Switzerland; ^6^Health Technology Assessment Unit, Academic Education, Research and Innovation Area, General Directorate, Ente Ospedaliero Cantonale, Bellinzona, Switzerland

**Keywords:** positron emission tomography/CT, molecular imaging, staging, lung cancer, small cell lung cancer

## Abstract

**Background:** Molecular imaging methods are currently used in the management of patients with lung cancer. Compared to non-small cell lung cancer, less data are available about the impact of molecular imaging using fluorine-18-fluorodeoxyglucose positron emission tomography/computed tomography (^18^F-FDG PET/CT) in staging patients with small cell lung cancer (SCLC). Performing a systematic review and meta-analysis, we aimed to provide quantitative data about the impact of ^18^F-FDG PET/CT in staging SCLC.

**Methods:** A comprehensive literature search of studies on the use of ^18^F-FDG PET/CT in patients with SCLC was performed. Three different databases were screened (PubMed/MEDLINE, EMBASE, and Cochrane library databases) until June 2019. Only articles describing the impact of ^18^F-FDG PET/CT in staging patients with SCLC were selected. A pooled analysis evaluating the change of binary SCLC staging (limited-stage vs. extensive-stage disease) using ^18^F-FDG PET/CT was carried out.

**Results:** Nine articles including 721 patients with SCLC were included in the systematic review. Compared to conventional staging, a superior diagnostic accuracy of ^18^F-FDG PET/CT was found. A change of binary SCLC staging using ^18^F-FDG PET/CT was demonstrated in 15% (95% confidence interval, 9–21%) of patients with SCLC. Currently, it is not clearly demonstrated that the use of ^18^F-FDG PET/CT for staging may improve the survival outcome of patients with SCLC.

**Conclusions:** ^18^F-FDG PET/CT is a useful molecular imaging method for staging patients with SCLC because it can change the management in a significant number of patients. More large prospective studies and cost-effectiveness analyses on the impact of ^18^F-FDG PET/CT in staging patients with SCLC are needed.

## Introduction

Neuroendocrine tumors are ~20% of all lung cancers, and small cell lung cancer (SCLC) is the most frequent neuroendocrine tumor of the lung. Most cases of SCLC are related to smoking, and the estimated incidence of this tumor in the United States for the year 2019 is 29,660 new cases/year with a male/female incidence ratio of 1:1 (1, 2).

SCLC is an aggressive high-grade neuroendocrine tumor characterized by rapid growth and early development of metastatic spread. Most SCLC patients have metastatic disease at the initial diagnosis, and about one-third of them has limited disease confined to the chest, whereas two-thirds of them has hematogenous metastases. Even if SCLC is usually highly sensitive to chemotherapy and radiation therapy, however, most patients develop recurrent disease (2, 3).

Most of the literature classifies SCLC patients based on a binary classification scheme to define the extent of the disease. Limited-stage disease (LD-SCLC) is confined to the ipsilateral hemithorax (including contralateral mediastinal and ipsilateral supraclavicular lymph nodal metastases) and can be included within a radiation field. Extensive-stage disease (ED-SCLC) is spread beyond the ipsilateral hemithorax (including hematogenous metastases and malignant pleural or pericardial effusion) (2–4). In patients with LD-SCLC, the standard treatment is usually chemotherapy plus thoracic radiation therapy. In this setting of patients, prophylactic cranial irradiation is also indicated to increase overall survival. In ED-SCLC, long-term survival is rare, and systemic therapy alone is considered a palliative treatment (2–4). Correct staging of SCLC is pivotal to assess the indication of thoracic radiation therapy, which is useful mainly in patients with LD-SCLC. Therefore, using a binary staging system in SCLC patients, only disagreement on the presence or absence of metastatic lesions outside one hemithorax or malignant pleural effusion will have a significant impact on patient management (4).

Computed tomography (CT) of chest and abdomen and brain magnetic resonance imaging (MRI) are the most used imaging methods for staging SCLC (4). However, if LD-SCLC is suspected, molecular imaging using fluorine-18 fluorodeoxyglucose positron emission tomography (^18^F-FDG PET) can be performed to assess for distant metastases. In particular, hybrid imaging using ^18^F-FDG PET/CT, providing both functional and morphological data, has been demonstrated to be superior to ^18^F-FDG PET alone, and it is the current state of art for molecular imaging of lung cancer (5). As SCLC is an aggressive neuroendocrine tumor with increased metabolism and ^18^F-FDG is a radiolabeled glucose analog, an increased uptake of this radiopharmaceutical is expected by SCLC lesions (6–8). Compared to non-small cell lung cancer, less data are available about the impact of molecular imaging using ^18^F-FDG PET/CT in staging patients with SCLC. Therefore, we aimed to provide timely evidence-based data on the use of this imaging method for staging SCLC.

## Methods

This evidence-based article was written according to the “Preferred Reporting Items for a Systematic Review and Meta-analysis of Diagnostic Test Accuracy Studies” (9–11). This review has been registered on the online database PROSPERO (international prospective register of systematic reviews) after submission, as requested by the reviewers.

### Review Question and Patient/Intervention/Comparison/Outcome Process

The review question was formulated according to the Patient/Intervention/Comparison/Outcome process:

- Patients: individuals with histologically proven SCLC;
- Intervention: ^18^F-FDG PET/CT performed for disease staging;
- Comparison: staging without ^18^F-FDG PET/CT (e.g., using conventional imaging methods such as contrast-enhanced CT or bone scintigraphy);
- Outcomes: the main outcome was the change of binary SCLC staging using ^18^F-FDG PET/CT; secondary outcomes were the diagnostic accuracy of ^18^F-FDG PET/CT staging compared to conventional staging in SCLC and the impact of ^18^F-FDG PET/CT staging on survival of SCLC patients.

### Search Strategy

Two authors (FM and GT) performed a comprehensive computer literature search of three different bibliographic databases (PubMed/MEDLINE, EMBASE, and Cochrane library) to find published articles on the impact of ^18^F-FDG PET/CT in staging patients with SCLC. A search algorithm combining several keywords related to the index test and the target condition was used: (A) “SCLC” and (B) “PET/CT” or “PET-CT.” No beginning date limit nor language restrictions were used. The literature search was performed until June 30th, 2019. To expand the literature search, we also screened the references of the retrieved articles searching for additional studies, which could be included in the systematic review.

### Study Selection

Inclusion criteria were studies or subsets of studies investigating the impact of ^18^F-FDG PET/CT in staging patients with SCLC histologically proved. The exclusion criteria were (a) articles outside the field of interest of this review (including articles using ^18^F-FDG PET only without hybrid technology for staging, studies evaluating the prognostic value of ^18^F-FDG PET/CT or its use for radiotherapy planning, and articles evaluating the role of ^18^F-FDG PET/CT for treatment response assessment or restaging after treatment); (b) review articles, letters, comments, editorials, and conference proceedings; and (c) case reports or small case series.

Two researchers (FM and GT) independently reviewed the titles and abstracts of the retrieved articles, applying the inclusion and exclusion criteria mentioned above. The same two researchers then independently reviewed the full text of the selected articles to assess their eligibility for inclusion. In cases of studies performing both ^18^F-FDG PET and PET/CT, whether sufficient information on the findings of the subgroup performing ^18^F-FDG PET/CT were not provided, we have excluded them from the qualitative analysis. A consensus meeting among the researchers was performed to solve any disagreement.

### Data Extraction

For each study included in the qualitative analysis, we collected the following information: authors, year of publication, country of origin, study design and level of evidence, patient characteristics (overall number of SCLC patients performing ^18^F-FDG PET/CT, mean age, sex ratio), technical aspects (hybrid imaging modality, injected radiopharmaceutical activity, time interval between radiopharmaceutical injection and image acquisition, image analysis, comparison with other imaging methods), diagnostic accuracy of ^18^F-FDG PET/CT for staging compared to conventional imaging (on a patient-based analysis), percentage of change of binary SCLC staging (from LD-SCLC to ED-SCLC and vice versa) using ^18^F-FDG PET/CT, and impact of ^18^F-FDG PET/CT on the survival outcome of SCLC patients.

### Quality Assessment

The overall quality of the studies included in the systematic review and meta-analysis was performed using the revised “Quality Assessment of Diagnostic Accuracy Studies” tool (12).

### Statistical Analysis

The change of binary SCLC staging by ^18^F-FDG PET/CT was defined as the ratio between the number of SCLC patients with a change of staging obtained by ^18^F-FDG PET/CT (upstaging from LD-SCLC to ED-SCLC or downstaging from ED-SCLC to LD-SCLC, respectively) and the overall number of SCLC patients who underwent the ^18^F-FDG PET/CT scan. Pooled analyses were performed using a random-effects model (taking into account the variability between studies). Pooled data were presented with their respective 95% confidence interval (95% CI) values and displayed using forest plots. Heterogeneity was estimated using the *I*-square index (*I*^2^) (13). Publication bias was assessed through the Egger's test (14). StatsDirect software version 3 (StatsDirect Ltd., Cambridge, UK) was used for the meta-analysis.

## Results

### Literature Search

**Figure 1:**
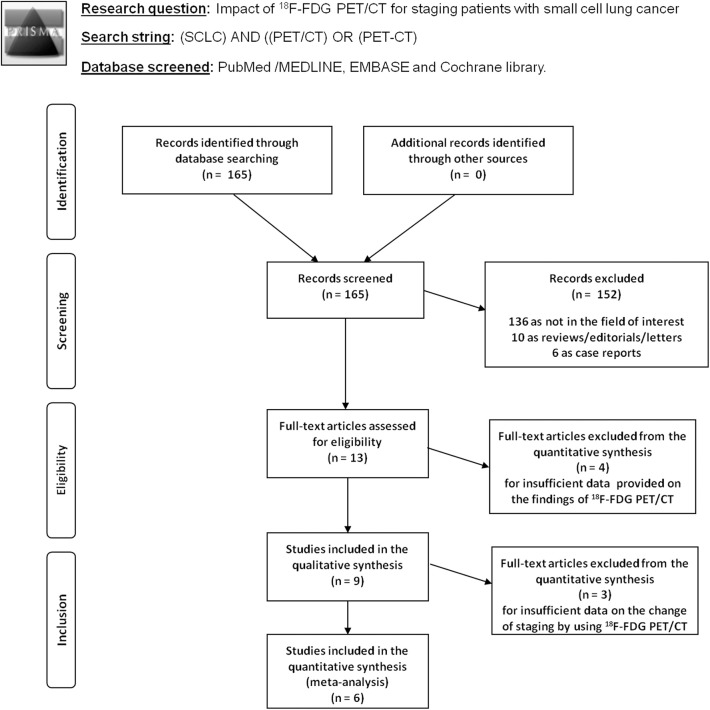
Flow chart of the search for eligible studies on the impact of fluorine-18-fluorodeoxyglucose positron emission tomography/computed tomography (^18^F-FDG PET/CT) for small cell lung cancer (SCLC) staging.

**Figure 2:**
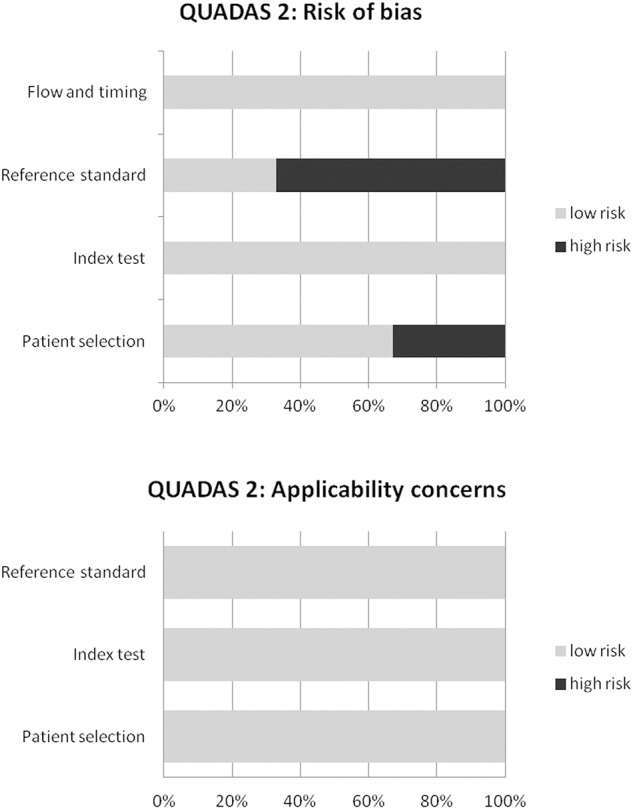
Overall quality assessment of the studies included in the systematic review according to the Quality Assessment of Diagnostic Accuracy Studies (QUADAS-2) tool.

The results of the literature search are reported in Figure 1. The comprehensive computer literature search revealed 165 articles. Reviewing titles and abstracts, 152 records were excluded: 136 because they were outside the field of interest of this systematic review; 10 as reviews, comments, editorials, or letters; and 6 as case reports. Thirteen articles were selected, and their full text was retrieved (15–27). No additional records were found screening the reference list of the retrieved articles. Four articles were excluded from the analysis because both ^18^F-FDG PET and PET/CT were performed, but sufficient information about the findings of the ^18^F-FDG PET/CT subgroup were not provided (15–18). Finally, nine articles including 791 patients who underwent ^18^F-FDG PET/CT were eligible for the systematic review (19–27). Six articles about change of binary SCLC staging using ^18^F-FDG PET/CT in 277 patients were included in the meta-analysis (6, 20–22, 24, 27). The characteristics of the included studies are presented in Tables 1–3. The overall quality assessment of these studies is reported in Figure 2.

**Table 1:** Basic study and patient characteristics on the impact of ^18^F-FDG PET/CT in staging patients with small cell lung cancer.

**References**	**Country**	**Study design**	**Level of evidence**	**No. of SCLC patients undergoing ^**18**^F-FDG PET/CT**	**Median age (years)**	**Male**
Manoharan et al. (19)	United Kingdom	Prospective multicenter	III	309	62	57%
Kishida et al. (20)	Japan	Prospective single center	III	59	69.6	86%
Saima et al. (21)	Pakistan	Retrospective single center	III	23	58	83%
Zer et al. (22)	Israel	Retrospective single center	III	55	66	65%
Xanthopoulos et al. (23)	USA	Retrospective single center	III	40	67	22%
Sohn et al. (24)	Korea	Retrospective single center	III	73	62	82%
Lee et al. (25)	Korea	Retrospective single center	III	95	68	75%
Niho et al. (26)	Japan	Retrospective single center	III	38	64	90%
Fischer et al. (27)	Denmark	Prospective single center	III	29	63	38%

*^18^F-FDG, fluorine-18 fluorodeoxyglucose; NR, not reported; PET/CT, positron emission tomography/computed tomography; PET/MRI, positron emission tomography/magnetic resonance imaging*.

**Table 2:** Technical aspects of ^18^F-FDG PET/CT in the included studies.

**References**	**Hybrid imaging modality**	**Mean injected activity**	**Time interval between radiotracer injection and image acquisition**	**Image analysis**	**Other imaging modalities used for comparison**
Manoharan et al. (19)	NR	NR	NR	NR	CT, bone scintigraphy
Kishida et al. (20)	PET/CT (low-dose CT)	5.18 MBq/kg	60 min	Visual	CT, bone scintigraphy
Saima et al. (21)	PET/CT (low-dose CT)	300 MBq	60 min	Visual and semiquantitative (SUV_max_)	CT
Zer et al. (22)	PET/CT (contrast-enhanced CT)	370–666 MBq	NR	Visual and semiquantitative (SUV_max_, MTV, TLG)	CT, bone scintigraphy
Xanthopoulos et al. (23)	NR	NR	NR	NR	CT, bone scintigraphy
Sohn et al. (24)	PET/CT (low-dose CT)	550 MBq	60 min	Visual and semiquantitative (SUV_max_)	CT, bone scintigraphy
Lee et al. (25)	PET/CT (low-dose CT)	5.18 MBq/kg	60 min	Visual	Bone scintigraphy
Niho et al. (26)	PET/CT (low-dose CT)	300 MBq	60 min	Visual	CT, bone scintigraphy
Fischer et al. (27)	PET/CT (contrast-enhanced CT)	400 MBq	60 min	Visual	CT, bone scintigraphy

*L^18^F-FDG, fluorine-18 fluorodeoxyglucose; CT, computed tomography; MBq, MegaBecquerel; min, minutes; MTV, metabolic tumor volume; NR, not reported; PET, positron emission tomography; SUV_max_, maximal standardized uptake value; TLG, total lesion glycolysis*.

**Table 3:** Data about impact of ^18^F-FDG PET/CT for staging SCLC.

**References**	**Sensitivity/specificity/PPV/NPV of ^**18**^F-FDG PET/CT staging (LD-SCLC vs. ED-SCLC)**	**Diagnostic accuracy of ^**18**^F-FDG PET/CT staging**	**Diagnostic accuracy of conventional staging**	**Change of binary stage by using ^**18**^F-FDG PET/CT**	**Improved outcome by using ^**18**^F-FDG PET/CT**
Manoharan et al. (19)	NR/NR/NR/NR	–	–	–	No
Kishida et al. (20)	NR/NR/NR/NR	57/59 (96.6%)	54/59 (91.5%)	3/59 (5.1%)	NR
Saima et al. (21)	100%/100%/100%100%	23/23 (100%)	18/23 (78.3%)	5/23 (21.7%)	NR
Zer et al. (22)	NR/NR/NR/NR	–	–	6/55 (10.9%)	NR
Xanthopoulos et al. (23)	NR/NR/NR/NR	–	–	–	Yes
Sohn et al. (24)	NR/NR/NR/NR	–	–	16/73 (21.9%)	NR
Lee et al. (25)	NR/NR/NR/NR	–	–	–	NR
Niho et al. (26)	NR/NR/NR/NR	–	–	5/38 (13.2%)	NR
Fischer et al. (27)	93%/100%/100%/86%	19/20 (95%)	17/20 (85%)	5/29 (17.2%)	NR

*ED-SCLC extensive-stage disease small cell lung cancer; LD-SCLC, limited-stage disease small cell lung cancer; NR, not reported; PET, positron emission tomography; PPV, positive predictive value; NPV, negative predictive value*.

### Qualitative Analysis (Systematic Review)

#### Basic Study and Patient Characteristics

Nine articles including data on the impact of ^18^F-FDG PET/CT in staging SCLC were selected (Table 1) (19–27). The included studies were published from 2007 to 2019. Patients from several countries and different continents were represented. One-third of the studies were prospective, and most of them were single-center studies (89%). The median age of the SCLC patients ranged from 58 to 69.6 years. The percentage of male patients widely ranged from 22 to 90%.

#### Technical Aspects

The technical aspects about ^18^F-FDG PET/CT were heterogeneous (Table 2). The hybrid imaging modality was PET/CT using low-dose CT in most of the cases and contrast-enhanced CT in 22% of the studies. Mean injected radiotracer activity were quite different among the included studies. The mean time between radiopharmaceutical injection and image acquisition was 60 min. The analysis of PET images was carried out using visual/qualitative analysis in all studies. In some studies, a further analysis using the calculation of semiquantitative parameters, for instance the maximal standardized uptake value (SUV_max_), was performed. A composite reference standard including histology, further imaging, or clinical/biochemical follow-up was used in the included studies. ^18^F-FDG PET/CT findings were compared with CT or bone scintigraphy findings in most of the articles.

#### Main Findings

Three studies evaluated the diagnostic accuracy of ^18^F-FDG PET/CT staging compared to conventional staging without ^18^F-FDG PET/CT in patients with SCLC (20, 21, 27).

In the prospective study of Fischer et al., the diagnostic performance of ^18^F-FDG PET/CT in correctly staging LD-SCLC and ED-SCLC was 95% (19 out of 20 SCLC correctly staged), and it was higher compared to that of conventional imaging methods (85%; 17 out of 20 patients correctly staged) (27). The better diagnostic performance of ^18^F-FDG PET/CT in correctly staging LD-SCLC and ED-SCLC was also confirmed by another prospective study of Kishida et al. who found a diagnostic accuracy of 96.6% (57/59 patients correctly staged as ED-SCLC or LD-SCLC) for ^18^F-FDG PET/CT compared to 91.5% (54/59 patients correctly staged as ED-SCLC or LD-SCLC) for conventional staging, even if this difference was not statistically significant (20).

Six studies assessed the change of binary SCLC staging using ^18^F-FDG PET/CT compared to conventional staging (upstaging from LD-SCLC to ED-SCLC or downstaging from ED-SCLC to LD-SCLC, respectively) reporting percentages ranging from 5.1 to 21.7% (20–22, 24, 26, 27).

About the comparison among ^18^F-FDG PET/CT and bone scintigraphy for SCLC staging, the study of Lee et al. demonstrated that, in patients with SCLC, ^18^F-FDG PET/CT showed higher detection rate of bone metastases than bone scintigraphy. In 95 SCLC performing both methods for detecting bone metastases, the sensitivity of ^18^F-FDG PET/CT was 100 and 87% on a per-patient- and on a per-lesion-based analysis, respectively; conversely, the sensitivity of bone scintigraphy was 37 and 29% on a per-patient- and on a per-lesion-based analysis, respectively. Based on these findings, ^18^F-FDG PET/CT should replace bone scintigraphy for staging SCLC (25).

On the other hand, for detecting brain metastases of SCLC, ^18^F-FDG PET/CT cannot substitute brain MRI due to the limited detection rate of brain metastases by ^18^F-FDG PET/CT (20); the main concern about the use of ^18^F-FDG PET for evaluating brain lesions is the high background ^18^F-FDG uptake in normal brain.

About the impact of ^18^F-FDG PET/CT staging on outcome of SCLC patients, the survival outcomes were similar in patients staged with or without ^18^F-FDG PET/CT, according to the recently published analysis of the CONVERT randomized controlled trial; however, this analysis cannot support the omission of ^18^F-FDG PET/CT for SCLC staging due to some study limitations, as reported by the authors (19).

Xanthopoulos et al. found that LD-SCLC patients staged with ^18^F-FDG PET/CT exhibited improved disease control and survival when compared with LD-SCLC patients staged without ^18^F-FDG PET/CT. The median overall survival from diagnosis in patients staged with ^18^F-FDG PET/CT was 32 vs. 17 months in patients staged without PET/CT (*p* = 0.03), and 3-year survival was 47 vs. 19%, respectively. Median time-to-distant failure was 29 vs. 12 months, respectively (*p* = 0.04); median time-to-local failure was not reached vs. 16 months, respectively (*p* = 0.04). On multivariable analysis, ^18^F-FDG PET/CT staging was associated with survival (odds ratio = 0.24; *p* = 0.04) (23).

### Quantitative Analysis (Meta-Analysis)

**Figure 3:**
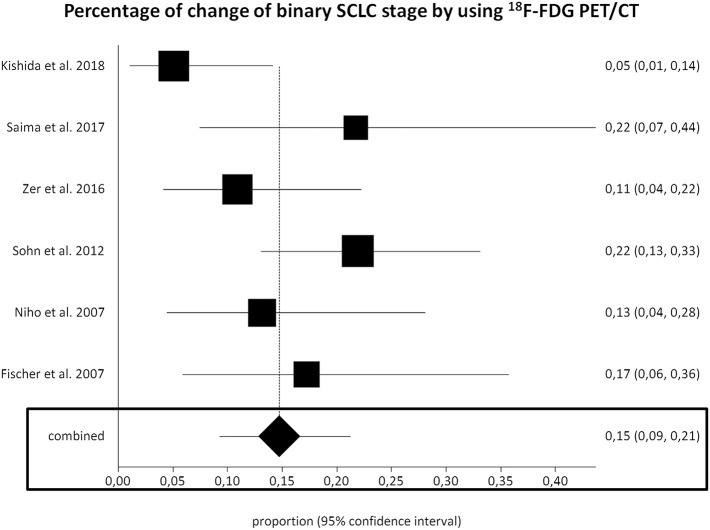
Plots of individual studies and pooled percentage of change of binary small cell lung cancer (SCLC) stage using fluorine-18-fluorodeoxyglucose positron emission tomography/computed tomography (^18^F-FDG PET/CT), including 95% confidence intervals (95% CI). The size of the squares indicates the weight of each study.

Six studies (277 SCLC patients) were selected for the meta-analysis on the change of binary SCLC staging using ^18^F-FDG PET/CT (20–22, 24, 26, 27). The pooled percentage of change of binary SCLC stage using ^18^F-FDG PET/CT (upstaging from LD-SCLC to ED-SCLC or downstaging from ED-SCLC to LD-SCLC, respectively) was 15% (95% CI, 9–21%) (Figure 3). A moderate heterogeneity among the included studies was found (*I*^2^ = 50%). The Egger's test did not demonstrate a significant publication bias (*p* = 0.08).

Performing a subgroup analysis based on the different type of study design, the percentage of change of binary SCLC staging using ^18^F-FDG PET/CT in prospective and retrospective studies was 11% and (95% CI, 2–25%) and 17% (95% CI, 12–23%), respectively.

## Discussion

This is the first meta-analysis focused on the impact of hybrid ^18^F-FDG PET/CT for staging of SCLC patients. In fact, previous published evidence-based documents included mostly articles about the use of ^18^F-FDG PET only in staging SCLC (28–32). As the current state of the art of PET imaging in oncology is hybrid ^18^F-FDG PET/CT, we have provided timely and updated information selecting only articles that have evaluated the impact of SCLC staging using this hybrid imaging modality.

Several studies have used ^18^F-FDG PET/CT for staging SCLC patients; however, most of these studies have limited statistical power, due to their relatively small patient population. We have pooled data from the published studies to provide more robust estimates on the impact of ^18^F-FDG PET/CT for staging SCLC patients.

The most relevant information provided by our systematic review and meta-analysis is that ^18^F-FDG PET/CT has an impact in staging SCLC patients, as this method may change the binary SCLC stage (from LD-SCLC to ED-SCLC or vice versa) in ~15% of patients. This change of binary SCLC stage automatically leads to a change of therapeutic management, as only LD-SCLC may have benefit of a radiation therapy for curative intent (4).

The change of binary SCLC stage may be related to the better diagnostic accuracy of ^18^F-FDG PET/CT compared to conventional imaging methods in detecting SCLC metastases in different organs (except for brain metastases), as functional abnormalities detected by ^18^F-FDG PET usually precede morphological alterations revealed by conventional imaging methods (5). To this regard, in a previous meta-analysis, the pooled sensitivity, specificity, and positive and negative likelihood ratio (LR+ and LR–) of ^18^F-FDG PET or PET/CT in diagnosing ED-SCLC were 97.5% (95%CI, 94.2–99.2%), 98.2% (95% CI, 94.9–99.6%), 19.86 (95% CI, 9.79–40.3), and 0.06 (95% CI, 0.03–0.1), respectively (29). There were no significant differences in diagnostic accuracy between ^18^F-FDG PET/CT and PET alone in the detection of ED-SCLC, but limited data about ^18^F-FDG PET/CT were available (29).

Current National Comprehensive Cancer Network guidelines suggest to perform ^18^F-FDG PET/CT for staging SCLC only if LD-SCLC is suspected (4). Furthermore, the American College of Radiology appropriateness criteria recommend the use of ^18^F-FDG PET/CT in patients with suspicious LD-SCLC being considered for treatment with curative intent, whereas the use of ^18^F-FDG PET/CT for further staging is considered optional if ED-SCLC is established (33). Evidence-based data provided by our meta-analysis strengthen the current recommendations on the use of ^18^F-FDG PET/CT for staging SCLC patients.

Some limitations of our analysis should be underlined. The first limitation is the low number of included studies on the impact of ^18^F-FDG PET/CT in staging SCLC. Another limitation is the heterogeneity among the included studies likely based on differences of patient characteristics, methodological aspects, and study quality. Conversely, we did not detect a significant publication bias in our meta-analysis.

Based on the data reported in our systematic review, we would like to suggest to perform more large multicentric and prospective studies on the impact of ^18^F-FDG PET/CT for SCLC staging to strengthen its role in this setting.

Furthermore, more prospective studies to clarify the impact of ^18^F-FDG PET/CT staging on the survival outcomes of SCLC patients would be useful to support its routine clinical use for SCLC staging. Unfortunately, the available data to this regard are not univocal (19, 23). The major advantages of ^18^F-FDG PET/CT staging in SCLC may lie in avoiding unnecessary toxicity in ED-SCLC and in suggesting the appropriate addition of thoracic radiation therapy in LD-SCLC with potential better patient survival (5). It has been recently demonstrated by a retrospective study in a mixed SCLC patient population performing both ^18^F-FDG PET and PET/CT that ^18^F-FDG PET staging in patients with SCLC was associated with greater overall survival and lung-cancer-specific survival, likely reflecting stage migration and stage-appropriate therapy (17).

Lastly, cost-effectiveness analyses on the use of ^18^F-FDG PET/CT in staging SCLC are currently lacking in the literature. One article performed a cost analysis in a European setting, taking into account the overall costs of staging and therapy of SCLC, using two different strategies for staging (staging with ^18^F-FDG PET/CT compared to conventional staging without ^18^F-FDG PET/CT), reporting a non-significant difference in overall costs among these strategies (27). Another cost analysis in an Australian setting demonstrated that the initial costs of the two staging strategies, with or without ^18^F-FDG PET/CT, were not significantly different. ^18^F-FDG PET/CT staging may reduce healthcare costs for SCLC patients through avoidance of inappropriate thoracic radiation therapy (31).

## Conclusions

^18^F-FDG PET/CT is a very useful imaging method in staging SCLC patients, as it may change the binary SCLC stage, resulting in change of therapeutic management, in ~15% of cases. Further well-designed studies on the use of this hybrid imaging method for SCLC staging are needed.

## Data Availability Statement

The datasets generated for this study are available on request to the corresponding author.

## Author Contributions

All authors contributed to the manuscript. Different tasks are described here below. FM and GT: conceptualization. GT and MP: methodology. FM, MP, MV, GP, PF, LG, and AR: data curation. FM and GT: writing. MV, GP, PF, LG, and AR: review and editing.

### Conflict of Interest

The authors declare that the research was conducted in the absence of any commercial or financial relationships that could be construed as a potential conflict of interest.
